# Comparison of Caffeoylquinic Acids and Functional Properties of Domestic Sweet Potato (*Ipomoea batatas* (L.) Lam.) Storage Roots with Established Overseas Varieties

**DOI:** 10.3390/foods11091329

**Published:** 2022-05-03

**Authors:** Charmaine J. Phahlane, Sunette M. Laurie, Tinotenda Shoko, Vimbainashe E. Manhivi, Dharini Sivakumar

**Affiliations:** 1 Phytochemical Food Network Research Group, Department of Crop Sciences, Tshwane University of Technology, Pretoria 0001, South Africa; charmainejacqueline@gmail.com (C.J.P.); shokot@tut.ac.za (T.S.); ManhiviveE@tut.ac.za (V.E.M.); 2 Agricultural Research Council-Vegetables, Industrial and Medicinal Plants (ARC-VIMP), Pretoria 0001, South Africa; slaurie@arc.agric.za

**Keywords:** *Ipomoea batatas* (L.) Lam., phytochemicals, antioxidant properties, caffeoylquinic acid, chemometrics

## Abstract

Root samples of sweet potato varieties originating from South Africa (‘Ndou’, ‘Bophelo’, ‘Monate’, and ‘Blesbok’), the USA (‘Beauregard’), and Peru (‘199062.1′) were analyzed using ultra-high-performance liquid chromatography-quadrupole time-of-flight mass spectrometry (UPLC/QTOF/MS) and chemometrics to characterize and compare the locally developed varieties with well-known established overseas varieties. The highest total phenol content was detected in ‘Bophelo’, followed by ‘Beauregard’ and Peruvian variety ‘199062.1’. The Orthogonal Projections to Latent Structures Discriminant Analysis (OPLS-DA) model classified the storage roots of six sweet potato varieties into two clusters. In the OPLS-DA scatter plot, one cluster, which included Peruvian variety ‘199062.1’, was separated from the others. L-tryptophan and 3-caffeoylquinic acid (CQA) showed variable importance in projection (VIP) scores greater than 1.5. Based on the OPLS-DA-S-plot, L-tryptophan separated the other varieties from Peruvian variety ‘199062.1’. Peruvian variety ‘199062.1’ contained higher concentrations of CQA (1,3-diCQA, 1,4-diCQA, 3,5-diCQA, 4,5-diCQA, 3-CQA, and 5-CQA) and 5-hydroxy-6-methoxycoumarin 7-glucoside than other varieties. Among all sweet potato varieties analyzed, Peruvian variety ‘199062.1′ showed the highest ferric reducing antioxidant power (2,2-diphenyl-1-picryl-hydrazyl-hydrate) free radical scavenging activity, and [2,2′-azinobis-(3-ethylbenzothiazoline-6-sulfonate)] scavenging activity. Among the local sweet potato varieties, ‘Bophelo’ has the greatest potential for commercialization as it is the richest source of CQA.

## 1. Introduction

Sweet potato (*Ipomoea batatas* (L.) Lam.) is a dicotyledonous plant that belongs to the family Convolvulaceae [1]. As a result of their versatility and adaptability, sweet potatoes are ranked globally as the seventh most popularly consumed food crop [2]. As most countries grow sweet potatoes exclusively for domestic consumption, the production shares traded in the world market are small [3]. Sweet potatoes play a key role as ‘indigenized’ root crops in South Africa [4], and due to their drought tolerance and contribution to food security, they are an important crop for smallholders [4]. Sweet potato roots and leaves contain phenolic compounds [5]. The total phenolic content of four varieties grown in the United States differed by between 45 and 103 mg gallic acid equivalents/100 g (GAE) [5,6]. The most predominant phenolic compounds found in sweet potato roots are caffeoylquinic acid (CQA) derivatives [7]; therefore, sweet potatoes are a good source of chlorogenic acid. CQA-derivatives have been widely studied for their benefits, including their hepatoprotective, antihistamine, hypoglycemic, antioxidant and antiviral properties [8]. Reportedly, hepatic mitochondrial dysfunction is associated with obesity, diabetes, and metabolic syndrome. Consuming foods high in antioxidants is therefore beneficial to consumers’ health [8]. The 5-CQA, 3-, 4-, and 4,5-diCQA derivatives showed promise for modifying mitochondrial function in hepatic cells [9]. Among 14 popular sweet potato varieties developed in Europe and the United States, 5-CQA and 3,5-diCQA were the most abundant phenolic acids, a purple-fleshed genotype had the highest amount of 3,5 dicaffeoylquinic acid (485.6 µg/g dry weight), and a white-fleshed ‘Quarter Million’ variety had the highest concentration of chlorogenic acid (422.4 µg g^−1^ dry weight) [10]. There are differences in the concentrations of derivatives of CQA between varieties [5]. Nutritionally, 100 g of sweet potatoes contains proteins (1.6 g), carbohydrates (20.1 g), the mineral potassium (337 mg), dietary fiber (3 g), vitamins (vitamin C, folate vitamin B6), a small amount of fat (0.1 g), and sodium (55 mg) [11], the amounts of which vary due to variety as well as genetic and environmental factors [12]. Regular consumption of diets containing 20–33 g of fiber per day was known to reduce the risk of colon cancer and regulate bowel movements [13]. Furthermore, baked orange sweet potatoes with skin (one cup 200 g) provide more than seven times the amount of β-carotene needed by an adult per day [11]. 

Among the developed sweet potato varieties by the Agricultural Research Council (ARC) in South Africa, the cream-fleshed variety ‘Blesbok’ is by far the most commercially utilized [14]. The ARC has focused its breeding efforts on improving high yields and dry matter content, as well as β-carotene content and drought resistance, to benefit farmers in rural Southern Africa [14]. The sweet potato varieties ‘Bophelo’, ‘Ndou’, and ‘Monate’ are now popular at the local market. Considering the critical role that phenols play in human health, it is crucial to understand the phenolic composition of locally developed sweet potato varieties compared to imported varieties from the USA and Peru. The USA variety ‘Beauregard’ is grown in a number of countries of the world as well as in South Africa, while ‘199062.1′ belongs to the International Potato center in Lima, Peru, which was introduced to Africa and is being grown in Mozambique and Ghana, but not in South Africa. 

However, the information is limited on the content of phenolic compounds in the roots of locally used sweet potatoes. The variety ‘Bophelo’ was recommended by Nyati et al. [15] for its high iron, zinc, and beta-carotene content in South Africa. Therefore, it is essential to profile the functional compounds in the roots of locally grown sweet potato varieties in order to understand the components and makeup of these compounds and their health benefits.

Therefore, the primary objective of our study was to characterize the phenolic compounds in the roots of locally produced sweet potato varieties compared with the well-established varieties from the United States of America (‘Beauregard’) and Peru (‘199062.1’). The second objective was to compare the antioxidant activities of the roots of domestic varieties, USA’s Beauregard, and Peruvian variety 199062.1.

## 2. Materials and Methods 

### 2.1. Chemicals 

The following chemicals were used in this study: anhydrous sodium sulphate, potassium persulphate, sodium carbonate, butylated hydroxytoluene, solvents (methanol, *N*-hexane, acetone, isopropyl alcohol, acetic acid, and acetonitrile), formic acid, 2.2′-diphenyl-1-picrylhydrazyl (DPPH), 2,2′ Azino-bis(3 ethylbenzothiazoline-6-sulphonic acid) ABTS, 2,4,6-Tris(2-pyridyl)-s-triazine (TPTZ), iron (III) chloride hexahydrate, the analytical standards (gallic acid, neochlorogenic acid, lutein, zeaxanthin, p-coumaric acid, chlorogenic acid, caffeic acid, β-carotene, ferulic acid, and quercetin 3 glycoside), sodium hydroxide, hydrochloric acid, ammonium hydroxide, Trolox, and Folin−Ciocalteu reagent. The chemicals used in this study were purchased from Sigma Aldrich, Johannesburg, South Africa.

### 2.2. Plant Material

**Figure 1:**
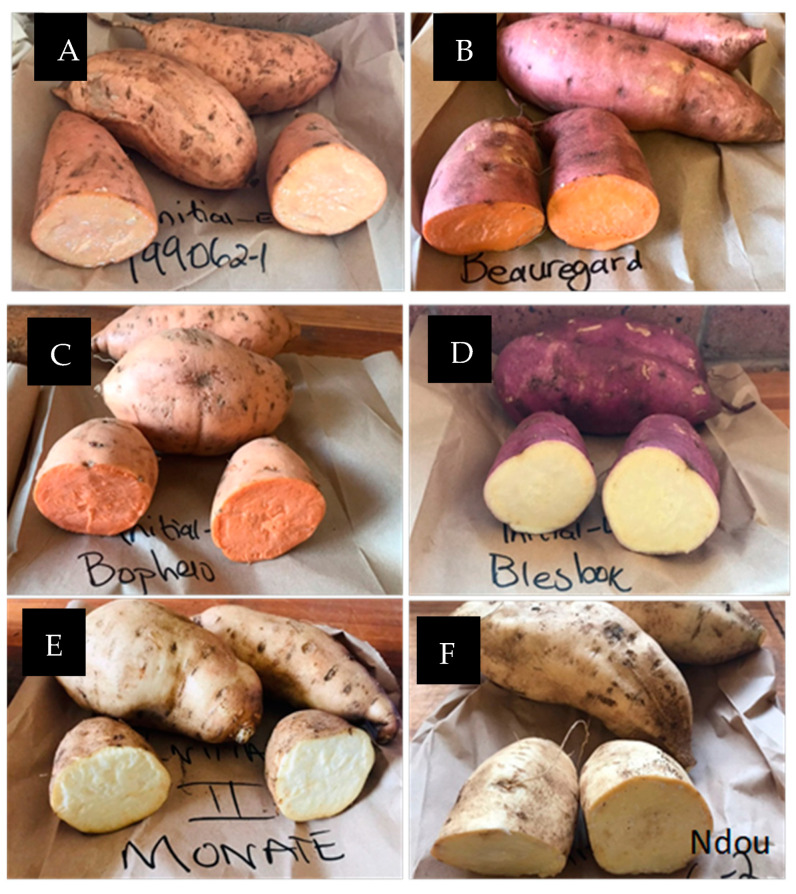
Illustrates the classification of the sweet potato varieties based on their flesh colour (**A**) Peruvian variety ‘199062.1’ (yellow orange flesh); (**B**) ‘Beauregard’ variety (USA, orange-fleshed); (**C**) ‘Bophelo’ (South Africa, orange-fleshed); (**D**) ‘Blesbok’ (South Africa, cream-fleshed); (**E**) ‘Monate’ (South Africa, cream-fleshed); and (**F**) ‘Ndou’ (South Africa, cream-fleshed.

The storage roots of orange-fleshed (‘Bophelo’) and three cream-fleshed varieties (‘Monate’, ‘Ndou’, and ‘Blesbok’) developed in South Africa as well as the USA’s ‘Beauregard’ variety (orange flesh) and Peru’s ‘199062.1′ variety (yellow-orange flesh) (average weight 210 g) (Figure 1) were obtained from the ARC-Vegetables, Industrial and Medicinal Plants (ARC-VIMP), Roodeplaat, Pretoria. As described by Phahlane et al. [16], the varieties were planted and replicated three times in a plot size of 7.2 m^2^ in mid-October 2020, at an average temperature of 25 to 31 °C, following standard production practices. After 4 months, the plants were harvested, followed by washing in tap water. Thereafter, the inner portion was retained after gently removing the outer periderm layer. In the inner portion, there was cortex (the outer layer of the cambium) and pith (the inner layer of the cambium). For biochemical analysis, the stem, middle, and bud end of the roots were cut into tiny slices and mixed well. Thereafter, the roots were freeze-dried (United Scientific model FM25XL-70 freeze dryer, −55 °C), ground into a powder, and held at −20°C.

### 2.3. Extraction

Freeze dried roots of each variety (1 g) were separately homogenized using 10 mL of 80:20 methanol/water (*v*/*v*) for 1 min; thereafter, the samples were sonicated (MRC Ultrasonic Cleaner, Model DC-150H, Company MRC Lab, Essex, UK) for 1 h and centrifuged at 2000× *g* using a centrifuge (Model Hermle Z326k, Hermle Labortechnik, Wehingen, Germany). Each sample was extracted three times in a similar way. The supernatants were collected and stored at −5 °C before analysis. 

### 2.4. Total Phenols 

The Folin−Ciocalteu method [17,18,19] was adopted to measure phenolic content. An aliquot (100 µL) of the extract was mixed with 200 µL of 10% Folin−Ciocalteu, then 800 µL of 7.5% Na_2_CO_3_. The reaction was left to stand for 1 h, and afterward each mixture was pipetted to the microplate and the absorbance was read at 736 nm using a spectrophotometer (SPECTROstar^®^ Nano, 601 0751. BMG LABTECH, Ortenberg, Germany). As a reference standard, chlorogenic acid was made at concentrations varying from 0 to 100 µg mL^−1^. Total phenolic content was expressed in mg of chlorogenic equivalent (CAE) per kg based on a dry weight (DW) basis.

### 2.5. Quantification of Different Phenolic Compounds

Using the technique of Mashitoa et al. [19], phenolic compounds were analyzed without any modifications. Using this supernatant, phenolic compounds in different varieties of sweet potatoes were characterized and quantified. Quantification and characterization of phenolic compounds were carried out using a Waters Synapt G2 Quadrupole time-of flight (QTOF) mass spectrometer (MS) hyphenated to a Waters Acquity ultra-performance liquid chromatograph (UPLC) (Waters, Milford, MA, USA). A Photodiode Array (PDA) detector was used, and afterward the eluate was passed on to the mass spectrometer; as such, there was collection of UV and MS spectra. The negative mode electrospray ionization was applied with a cone voltage of 15 V, a desolvation temperature of 275 °C, and desolvation gas at 650 L h^−1^. Data were acquired by scanning from *m*/*z* 150 to 1500 *m*/*z* in resolution mode as well as in MSE mode. In MSE mode, two channels of MS data were acquired: one at a low collision energy (4 V) and the second using a collision energy ramp (40–100 V) to obtain fragmentation data as well. Leucine enkephalin was used as reference mass for accurate mass determination and the instrument was calibrated with sodium formate. In addition, a Waters HSS T3, 2.1 × 100 mm, 1.7 μm column was used, and the injection volume was 2 μL with the mobile phase consisting of 0.1% formic acid (solvent A) and acetonitrile containing 0.1% formic acid as solvent B. The gradient started at 100% solvent A for 1 min and changed to 28% B over 22 min in a linear way. It then went to 40% B over 50 s and a wash step of 1.5 min at 100% B, followed by re-equilibration to initial conditions for 4 min. The flow rate was 0.3 mL min^−1^, and the column temperature was maintained at 55 °C. 

Compounds were tentatively identified based on comparison of mass fragmentation data with that of known compounds in mass bank libraries. Quantification of different phenolic compounds was conducted based on peak area relative to the reference standards; chlorogenic acid, catechin, and rutin, which were injected at a concentration range of 0 to 100 µg mL^−1^ phenolic compounds, were quantified in mg kg^−1^ of dried plant material.

### 2.6. Antioxidant Properties

Ferric Reducing Antioxidant Power (FRAP) assay was conducted in accordance with Seke et al. [18]. FRAP reagent (150 µL) was pipetted into an aliquot (20 µL) of the extract. Thereafter, the mixture was incubated for 10 min. FRAP reagent contained 10 mmol·L^−1^ of TPTZ in 40 mM of HCl, and 20 mM FeCl_3_·6H_2_O in 20 mM of acetate buffer (pH 3.6) mixed in a 1:1:10 ratio, respectively. Afterward, the absorbance was read at 593 nm using a spectrophotometer. Trolox solution ranging from 0–30 mM was prepared to construct the calibration curves, and the antioxidant power was expressed in mM TEAC g^−1^. 

Following the procedure described by Seke et al. [18], the 2,2′-diphenyl-1-picrylhydrazyl radical scavenging ability assay (DPPH) was performed with slight changes. The freeze-dried sample (0.1 g) was mixed with methanol:water (80:20) (1 mL). The resulting sample mixture was centrifuged at 3000× *g* for 5 min at 4 °C using a centrifuge (Model Hermle Z326k, Hermle Labortechnik, Wehingen, Germany). Different sample concentrations (100 µL) were made by serial dilution (0–10 mg mL^−1^), and 200 µL DPPH solution (13 µL DPPH mL^−1^ methanol) was pipetted into each well. Thereafter, the set up was held for 20 min at 25 °C, and the absorbance was read at 517 nm. Based on the following equation, we determined the % inhibition:

DPPH% Inhibition = (A_0_ − A_1_/A_0_) × 100, where A_0_ is the absorbance of the DPPH radical solution and A_1_ is the absorbance of the sample.

DPPH radical elimination was given in percentage of antioxidant activity, and the inhibition percentage versus concentration chart was used to calculate the IC_50_.

2,2′-azino-bis (3-ethylbenzothiazoline-6-sulfonic acid) (ABTS^+^) radical scavenging was conducted according to Seke et al. [18]. ABTS radical cation (ABTS^+^) was produced by allowing the reaction between 7 mM ABTS stock solution with 4.9 mM potassium persulphate at a 1:1 ratio and incubating the mixture at 25 °C for 12–16 h prior to use. An aliquot of 40 µL of the sample (different concentrations from 0–10 mg mL^−1^ made by serial dilution) was pipetted into 200 µL of ABTS^+^. The mixture was incubated at 37 °C for 10 min in the dark; a decrease in absorbance at 734 nm was measured.

The % inhibition was determined according to the following equation:

ABTS% Inhibition = (A_0_ − A_1_/A_0_) × 100

where A_0_ is the absorbance of the ABTS radical solution and A_1_ is the absorbance of the sample. The IC_50_ (mg mL^−1^) was calculated from the graph of the inhibition percentage versus the concentration. 

### 2.7. Statistical Analysis 

The experiments were laid out in a completely randomized design with 10 replicates per variety, and the roots were harvested twice, namely in December 2019 and January 2020. The Genstat (VSN International, Hemel Hempstead, UK) for Windows 13th Edition (2010 version) analyzed the differences between the roots of different sweet potato varieties using a one-way ANOVA. In order to compare the means of the different biochemical components from the roots, ANOVA with Tukey’s honestly significant difference (HSD) post hoc test was used with *p* < 0.05. A set of three replicate samples of sweet potato roots per variety were analyzed by UPLC-Q-TOF/MS and imported into MetaAnalyst 5.0 for Orthogonal Projections to Latent Structures Discriminant Analysis (OPLS-DA) variable importance in projection (VIP) and heat maps.

## 3. Results and Discussion

### 3.1. Total Phenols 

**Figure 2:**
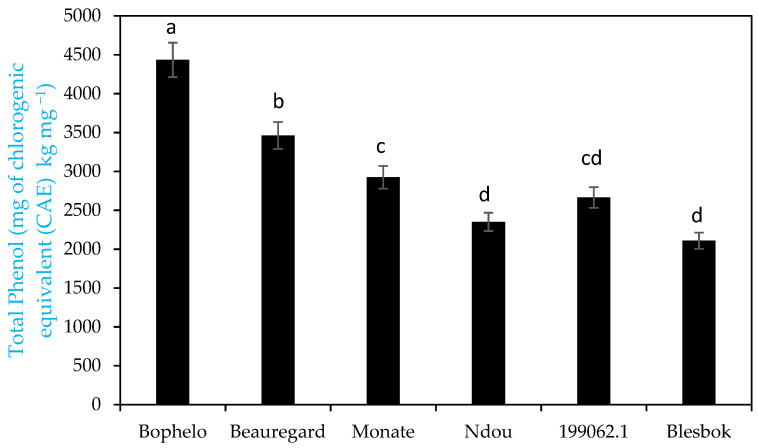
Comparison of total phenolic content in the storage roots of Southern African sweet potato (*Ipomoea batatas* L.) varieties with ‘Beauregard’ from the USA and Peruvian ‘199062.1′. Bars with the same letters of the alphabet are not significantly different at *p* < 0.05.

Other authors reported that the total phenolic content, the components of phenolic compounds, and the type of phenols vary widely, and sweet potato varieties differ in their antioxidant properties [20]. The total phenol content was highest in orange-fleshed storage roots ‘Bophelo’ (4434.44 mg kg^−1^ CAE ± 0.56), followed by ‘Beauregard’ (3462.22 mg kg^−1^ CAE ± 0.84) and cream-fleshed Monate (2925.18 mg kg^−1^ CAE ± 0.78) compared to ‘Ndou’, ‘Blesbok’, and Peruvian variety ‘199062.1’ (Figure 2). The level of total phenolic content present in ‘Bophelo’ was more or less similar to the levels found in Chinese varieties (4400 mg kg^−1^) [21]. Padda and Picha [9] found that when chlorogenic acid was used instead of gallic acid as a standard for the construction of standard curves, the level of total phenolics was higher. Storage roots of varieties such as ‘02-814’ (4700 mg kg^−1^, the USA’s purple-fleshed line) and ‘Quarter Million’ (4700 mg kg^−1^ CAE, Jamaica’s white-fleshed variety) showed slightly higher total phenolic content than that of ‘Bophelo’ on a dry weight basis.

### 3.2. Untargeted Metabolitle Profile

Twelve compounds were tentatively identified in the roots of six sweet potato varieties using UPLC-QTOF/MS, which were similar to the compounds characterized in the leaves of these same varieties in our previous investigation [16] (see Supplementary Figure S1: ESI negative mode BPI chromatogram of metabolites observed in the storage root of six different sweet potato varieties). The storage roots of six sweet potato varieties contained caffeoylquinic acid derivatives (neochlorogenic acid 5-CQA, chlorogenic acid 3-CQA, 3,5-dicaffeoylquinic acid (3,5-diCQA), 1,3-dicaffeoylquinic acid (1,3-diCQA), 1,4-dicaffeoylquinic acid (1,4-DCQA), and 4,5-dicaffeoylquinic acid (4,5-diCQA)), neoeriocitrin (eriodictyol 7-O neohesperidoside), caffeic acid, quercetin 3-glucosyl-(1->2)-galactoside, quercetin derivates, quercetin 3-galactoside, and quercetin-3-O-rutinoside (rutin) according to the UPLC–QTOF/MS technique. The presence of chlorogenic acid, caffeic acid, 4,5-diCQA, and 3,5-diCQA was also reported in 14 commercially important sweet potato genotypes for the European market [9]. Despite this, 3,4-dicaffeoylquinic acid (3,4-diCQA), which was found in the 14 genotypes previously reported by Padda and Picha [9], was not detected in the present study in the four locally developed varieties and the USA’s Beauregard and Peruvian varieties. Additionally, the roots of eight Korean varieties (‘Biomi’, ‘Shingeonmi’, ‘Shinhwangmi’, ‘Shinjami’, ‘Yeonhwangmi’, ‘Yulmi’, ‘Borami’, and ‘Yeonmi’) contained 3,4-diCQA [5]. The observed variation might be due to the methanol/water (80:20, *v*/*v*) solvent used in the extraction of derivatives of caffeoylquinic acids. However, Usuki et al. [21] reported that the use of 1-butyl-3-methylimidazolium chloride ([C4mim] Cl) allowed the extraction of 6.5-fold more CQAs than methanol. Further, environmental conditions in which the sweet potatoes were grown may have contributed to the absence of 3,4 diCQA in the locally grown varieties. In addition to tryptophan, terpene glycoside and S-nerolidol3-O-[a-L-rhamnopyranosyl-(1->4)-a-L-rhamnopyranosyl-(1->2)- 246 b-D-glucopyranoside] were detected in a methanol/water extract of sweet potato roots using UPLC-QTOF/MS technique.

### 3.3. Metabolomic and Chemometric Profiles 

**Figure 3:**
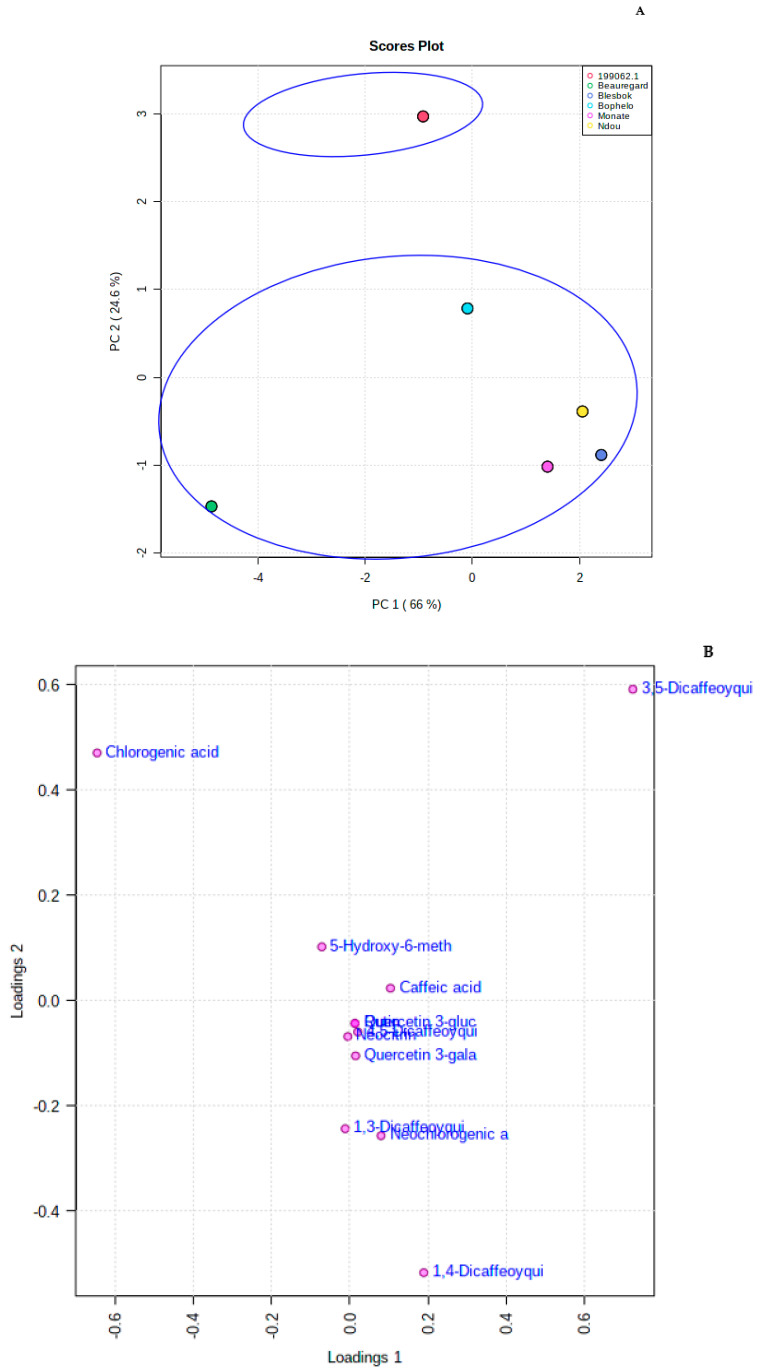

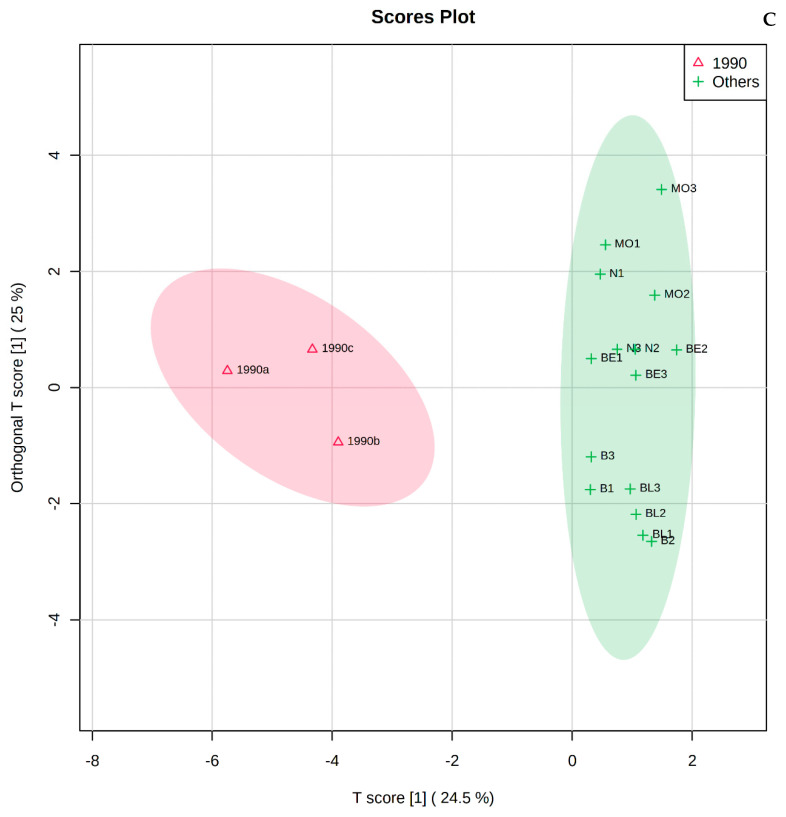

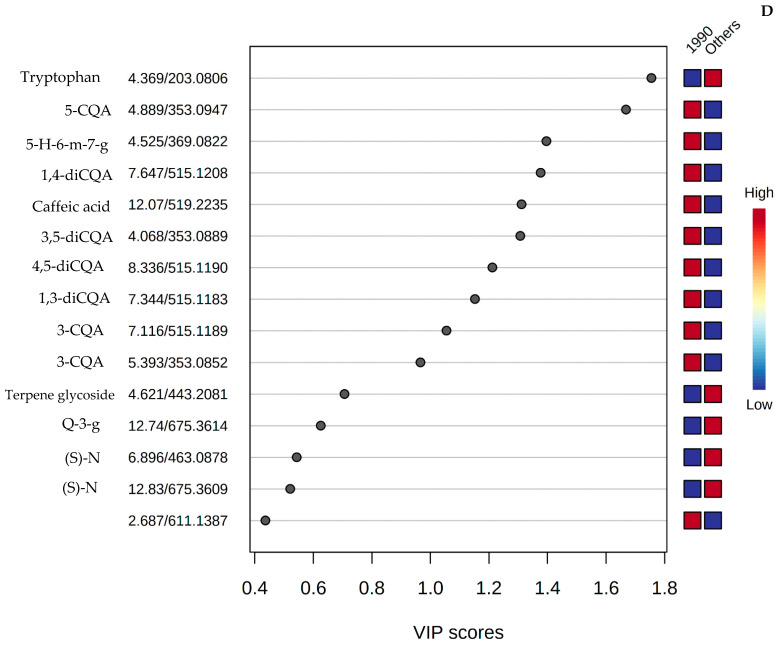

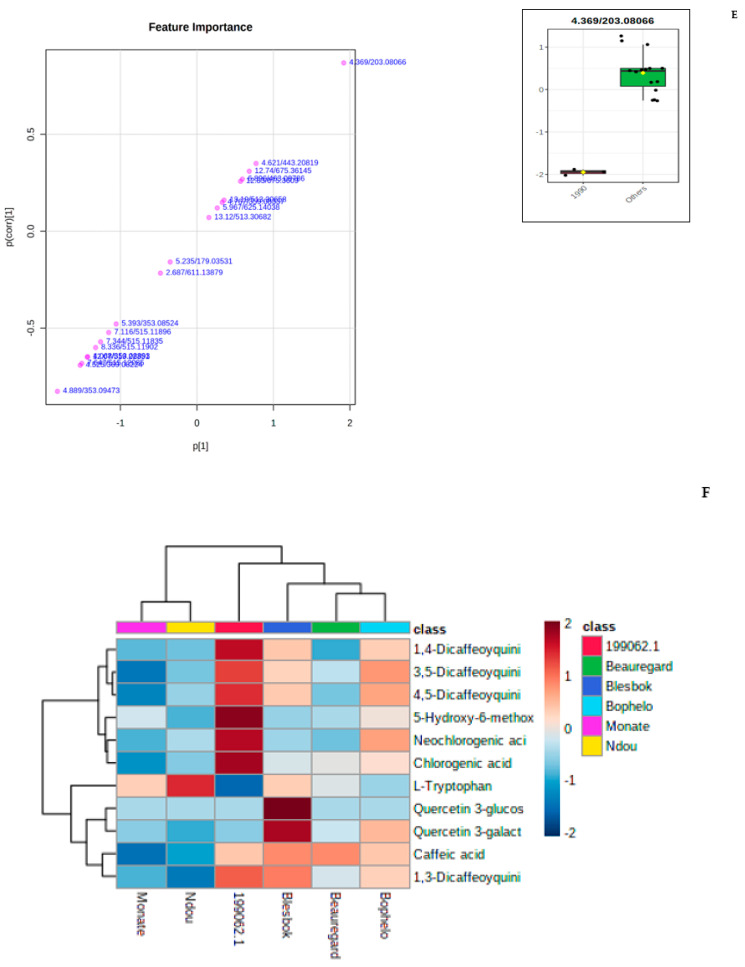
(**A**) Principal component analysis score plot showing the roots of six sweet potato 301 varieties segregated into two groups. The Peruvian variety ‘199062.1’ was separated from the 302 five sweet potato varieties (four local and ‘Beauregard’, (**B**). Principal component analysis score plots constructed with different metabo- 306 lites including phenolic compounds and amino acid detected by UPLC-QTOF-MS. The 307 phenolic compounds 3,5-diCQA and 3-CQA were responsible for the separation of Peruvian 308 variety ‘199062.1’ from the five sweet potato varieties (four local and ‘Beauregard’), (**C**). Orthogonal Projections to Latent Structures Discriminant Analysis score plot 312 constructed with different metabolites including phenolic compounds and amino acid de- 313 tected by UPLC-QTOF-MS showing roots of six sweet potato varieties clustered into two 314 groups separating the Peruvian variety ‘199062.1’ from the rest. 315 1990- Peruvian variety ‘199062.1’; MO- Monate; N-Ndou; BE- Beauregard; BL-Blesbok; B- 316 Bophelo 3, (**D**) In Partial Least-Squares Discriminant Analysis, metabolites including phenolic 320 compounds and amino acid were assigned VIP scores. 321 Scores indicate the importance of compounds and are illustrated in a pictorial man- 322 ner based upon the relative concentration of each compound. Dark red indicates maxi- 323 mum levels, while light blue indicates minimum levels. 324 5-Hydroxy-6-methoxycoumarin 7-glucoside = 5-H-6-m-7-g 325 (S)-Nerolidol 3-O-[a-L-Rhamnopyranosyl-(1->4)-a-L-rhamnopyranosyl-(1->2)-b-D- 326 glucopyranoside = (S)-N 327 Quercetin 3-galactoside = Q-3-g, (**E**). Orthogonal Projections to Latent Structures Discriminant Analysis -S-plot, showing the marker candidate L-tryptophan 332 separating the other varieties from Peruvian ‘199062.1’, (**F**) Heat map showing that all CQA derivatives and 5-hydroxy-6-methoxycouma- 337 rin 7-glucoside expressed at higher concentrations in the roots of Peruvian ‘199062.1’ com- 338 pared to other varieties investigated. The intensity of red color in the blocks relate to the 339 higher concentrations of the compounds. Dark red color means the concentration of a spe- 340 cific compound is very high. Light blue relates to the low intensity of a specific compound.

UPLC-Q-TOF/MS and unsupervised principal component analysis (PCA) technique allowed the separation of sweet potato varieties based on their phenolic compounds (Figure 3A). Principal component 1 and principal component 2 explained 90.6% of the variance (66.0% and 24.6%, respectively). In Figure 3B, different phenolic compounds of sweet potato roots were loaded onto the principal component analysis (PCA). As shown in Figure 3A, two primary groups or clusters of sweet potato varieties were prominent in a systematic and obvious manner based on their storage root phenolic compounds, separating Peruvian variety ‘199062.1′ from the rest. Peruvian variety ‘199062.1’ is distinguished from the rest by its 3,5-diCQA and 3-CQA (Figure 3B). Interestingly, these patterns of segregation or separation of clusters or groups indicate that different phenolic compounds play a significant role in defining clusters or groups of sweet potato roots.

According to Figure 3B, the farther away a point is from its original point, the more impact the compound has on the total variation. Principal component 2 shows positive loading of 3-CQA and 3,5-diCQA; further information needs to be extracted from the data, though, in order to identify and provide more specific and meaningful results. Thus, the UPLC-Q-TOF/MS was used for OPLS-DA analysis to determine changes in the metabolites based on the sweet potato variety. OPLS-DA builds a regression model between the multivariate data and a response variable that only contains class information. A good fit of the OPLS-DA model has been observed (R^2^ = 0.70), and the predictability of the model (Q^2^ = 0.60) predicted the changes of phenolic compounds from the data. Score scatter plots resulting from the OPLS-DA model are shown in Figure 3C. OPLS-DA analysis is a more accurate approach for prediction and descriptive modeling as it relies on no given distribution (Lee, Liong, and Jemain, 2018). According to the OPLS-DA model, storage roots of six sweet potato varieties were segregated into two clusters based on the untagged phenolic compounds. One cluster, which included the Peruvian variety ‘199062.1’, was separated from the others on the OPLS-DA scatter plot. PLS-DA also identifies which attributes (metabolites) score the highest in terms of VIP (Figure 3D). Thus, we assessed the aiding of each phenolic compound to the segregation of the groups using VIP scores. VIP scores are calculated by summing the squares of the PLS-DA loadings, which portray how much variance across all dimensions is explained by the PLS-DA loadings, and the weighted sum of the PLS-DA regression coefficients [22]. Thus, the phenolic compounds were ranked by VIP scores, and to provide the most substantive interpretation of the results, only the compounds with the highest VIP scores were included [23]. Unique metabolites with the topmost VIP scores (>1.5) were meticulously weighed to provide the most purposeful explanation of the results. Among the uppermost metabolites with VIP scores greater than 1.5 were l-tryptophan (1.67) and 5-CQA (1.78). Based on the OPLS-DA-S-plot, the amino acid L-tryptophan separated the other varieties from Peruvian variety ‘199062.1’ (Figure 3E); meanwhile, 3-CQA was used to separate Peruvian variety ‘199062.1’ from the other varieties. Phenolic compounds were used as biochemical markers that distinguish cultivars’ phenolic compounds [24]. In our previous investigations, we reported that the caffeic acid separated leaves of varieties ‘Beauregard’ and ‘Ndou’ from leaves of ‘199062.1’, ‘Bophelo’, ‘Monate’, and ‘Blesbok’.

In a heatmap, the levels of over- and under-expression of metabolites are compared, and a matrix is generated for each root variety. Moreover, heat maps can reveal patterns and groupings that are otherwise invisible. Heat map structures were generated for all samples based on the concentrations of metabolites. It is evident via the heat map (Figure 3F) that all CQA derivatives and 5-hydroxy-6-methoxycoumarin 7-glucoside were expressed at higher concentrations in Peruvian variety ‘199062.1’ than the other varieties investigated in this study.

### 3.4. Quantified Concentrations of Phenolic Compounds

Table 1 provides the concentrations of the different phenolic compounds present in each of the sweet potato varieties. It is evident from Table 2 that the derivatives of CQA (1,3-diCQA (31.97 ± 0.26 mg kg^−1^), 1,4-diCQA (21.40 ± 0.93 mg kg^−1^), 3,5-diCQA (36.77 ± 0.63 mg kg^−1^), 4,5-diCQA (0.86 ± 0.08 mg kg^−1^), 3-CQA (35.14 ± 0.45 mg kg^−1^), and 5-CQA (9.60 ± 0.23 mg kg^−1^) and 5-hydroxy-6-methoxycoumarin 7-glucoside (1.04 ± 0.05 mg kg^−1^) were detected at the highest concentrations in Peruvian variety ‘199062.1’ compared to the other varieties. Moreover, the roots of the local variety ‘Bophelo’ 0.70 ± 0.11 (mg kg^−1^) also showed a similar concentration of 4,5-diCQA as the Peruvian variety ‘199062.1’ (0.86 ± 0.08 mg kg^−1^). Among the local sweet potato varieties, ‘Bophelo’ is the richest source of CQA and other identified phenolic compounds. Furthermore, the concentrations of derivatives of CQA were higher in the storage roots of ‘Bophelo’ than in the USA’s ‘Beauregard’ variety. In contrast, CQA derivatives were detected at higher concentrations in the roots of ‘Blesbok’ than in those of ‘Ndou’ and ‘Monate.’ Individual phenolic components differed significantly among sweet potato varieties, as reported by Padda and Picha [9]. In general, 3-diCQA, 1,3-diCQA, and 3,5-diCQA were the richest CQAs found in all varieties. Similarly, Padda and Picha [9] noted that 3CQA and 3,5-dicaffeoylquinic acid were dominant CQAs in 14 different potato varieties traded in the USA and Europe. CQAs are consumed by consumers as milligram-to-gram proportions in healthy diets. CQAs have anti-inflammatory, antioxidant, and memory-improving properties that make them beneficial to consumers. Our results on the concentration of different CQA components in some instances cannot be compared with other varieties since the analysis was carried out on a dry weight basis. Further, the chlorogenic and dicaffeoylquinic acid contents of our results differed from the previously analyzed genotypes of orange- or cream-fleshed sweet potatoes, which may be due to a variety of factors, including different environmental conditions and different extraction conditions and techniques, among other factors [9].

### 3.5. Antioxidant Properties

Table 2 compares the antioxidant activities of four sweet potato varieties from South Africa with those of Beauregard and the ‘199062.1’ varieties from the USA and Peru. Peruvian variety ‘199062.1′ (532.06 µM TEAC g^−1^ ± 7.87) had the highest antioxidant power (FRAP) compared to all local varieties (184.20 to 525.63 µM TEAC g^−1^) and the USA’s ‘Beauregard’ (459.53 µM TEAC g^−1^ ± 1.84) variety. Moreover, the storage roots of the local variety ‘Bophelo’ (4.27 IC_50_ mg mL^−1^ ± 0.10), the USA’s ‘Beauregard’ variety 4.42 (IC_50_ mg mL^−1^ ± 0.03), and Peruvian variety ‘199062.1’ (4.60 IC_50_ mg mL^−1^) exhibited the maximum DPPH scavenging activity. Similarly, the ‘Bophelo’ (4.32 IC_50_ mg mL^−1^ ± 0.20), Beauregard (4.32 IC_50_ mg mL^−1^ ± 0.20), and ‘199062.1′ varieties (4.73 IC_50_ mg mL^−1^ ± 0.5) showed higher ABTS activities, while the local varieties ‘Monate’, ‘Ndou’, and ‘Blesbok’ showed the lowest DPPH and ABTS scavenging activities. Therefore, of all the analyzed storage roots of sweet potato varieties for antioxidant properties, Peruvian variety ‘199062.1′ showed the maximum FRAP, DPPH, and ABTS activities. In comparison to DPPH, which normally takes time to complete the reaction, ABTS is soluble in all solvents, and it acts swiftly [25]. As a result of color interference, DPPH assays underestimate antioxidant activity using samples containing anthocyanins. However, this complication does not occur when the ABTS method is used, especially when 734 nm absorbance is measured [25,26]. However, several investigators have used the DPPH method to detect the antioxidant properties of sweet potatoes [25,27,28]. Furthermore, the observed differences in the antioxidant properties between the different varieties can be attributed to the different antioxidant components present in theses varieties [16]. 

Prada and Picha [28] established a correlation coefficient (r^2^) between antioxidant activity and phenolic content of roots. Furthermore, Padda et al. [28] demonstrated that the antioxidant activity of sweet potato roots is reliant on root size, where a reduction in total phenolic content upon the growth of potato storage root is related to a dilution effect as a result of an increase in storage root weight. Additionally, the level of antioxidant activity will vary according to geographical location. Phenolic content and antioxidant properties are likely to be increased in plants that are grown in full sun and at high temperatures [29]. Strong antioxidants hold phenolic groups or a large number of conjugated hydroxyl groups that can donate electrons to oxidizing radical species [30]. In the presence of antioxidant molecules, cellular damage and macromolecular deterioration can be prevented [30]. Lebot et al. [30] showed that although the white- and orange-fleshed varieties lack anthocyanins, their antioxidant capacity is due to their higher CQA content. Lebot et al. [30] also showed that DPPH scavenging activity was positively correlated with CQA by examining sweet potatoes with different flesh colors. The total phenol concentration and antioxidant power (FRAP) were strongly and positively correlated (r^2^ = 0.76, *p* < 0.05) in the present study. The 5-CQA correlated to the FRAP activity (r^2^ = 0.75, *p* < 0.05), DPPH scavenging activity (r^2^ = 0.68, *p* < 0.05), and ABTS activity (r^2^ = 0.59, *p* < 0.05). Similarly, 3,5-diCQA correlated to the FRAP activity (r^2^ = 0.58, *p* < 0.05), DPPH scavenging activity (r^2^ = 0.50, *p* < 0.05), and ABTS activity (r^2^ = 0.52, *p* < 0.05). 1,3-diCQA showed a moderate correlation with FRAP activity (r^2^ = 0.50, *p* < 0.05), DPPH scavenging activity (r^2^ = 0.48, *p* < 0.05), and ABTS activity (r^2^ = 0.49, *p* < 0.05). Likewise, 3-CQA also showed a moderate correlation with FRAP activity (r^2^ = 0.52, *p* < 0.05), DPPH scavenging activity (r^2^ = 0.50, *p* < 0.05), and ABTS activity (r^2^ = 0.54, *p* < 0.05).

Therefore, 5-CQA, 3,5-diCQA, 1,3-diCQA, and 3-CQA were responsible for the observed antioxidant activity in theses varieties. According to Islam et al. [29], the presence of correlation between total phenolics and derivatives of the CQA will aid in the improvement of desirable parameters during cultivar selection or breeding. In a study by Kim et al., 2011, dicaffeoylquinic acids were found to have higher antioxidant activities—for example, DPPH, ABTS, and FRAP—than chlorogenic acid. Despite differing bonding positions, the antioxidant activity of dicaffeoylquinic acids (1,4, 1,3, 3,5, and 4,5 positions) was similarly based on their structures. On the other hand, according to the structure, 5 hydroxy-6 methoxycoumarin 7 glucoside may exhibit lower antioxidant activity in tests such as DPPH, ABTS, etc., because the methoxy group tends to remove electrons and reduces antioxidant activity. 

Furthermore, according to Cho et al. [31,32], higher antioxidant activity was associated with the presence of catechol groups, and since dicaffeoylquinic acids contained two catechol groups, they were found to be more powerful antioxidants. Since flavonoids (quercetin 3 glucosyl-(1->2)-galactoside, quercetin 3 galactoside, and rutin, a quercetin derivative) have a catechol structure in the B ring, it is expected that they have a high antioxidant activity like that of dicaffeoylquinic acids (Kim et al., 2011). However, in this study, quercetin 3 glucosyl-(1->2)-galactoside, quercetin 3 galactoside, and rutin were accumulated at lower concentrations in the roots of all six varieties of sweet potatoes (Table 1), while all four CQA derivatives except 1,4-diCQA were accumulated at higher concentrations in the roots of all six varieties (Table 1), which could be the possible reason for the observed positive and moderate correlations. 

## 4. Conclusions 

When comparing four local sweet potato varieties, ‘Beauregard’ from the USA, and ‘199062.1′ from Peru for antioxidant properties, Peruvian variety ‘199062.1′ showed the maximum antioxidant properties (FRAP, DPPH, and ABTS activities). Among the local sweet potato varieties, ‘Bophelo’ showed the highest antioxidant activity, the richest source of CQA, and other identified phenolic compounds, and these concentrations were higher than in the USA’s ‘Beauregard’ while being in the range of Peruvian ‘199062.1’. Thus, the local sweet potato variety ‘Bophelo’ can be recommended to produce functional products. In addition, the 5-CQA showed a positive correlation between antioxidant activities. Tryptophan and 5-CQA can also be used as markers to distinguish the roots of Peruvian variety ‘199062.1’ from the other five varieties included in this study.

## Figures and Tables

**Table 1:** Different phenolic components present in the storage roots of Southern African sweet potato (*Ipomoea batatas* L.) varieties compared with ‘Beauregard’ from the USA and Peruvian ‘199062.1′.

Phenolic Compounds (mg kg^−1^)	Bophelo	Beauregard	Monate	199062.1	Ndou	Blesbok
Neochlorogenic acid (5-CQA)	6.39 ± 0.23 b	1.91 ± 0.02 c	1.46 ± 0.43 c	9.60 ± 0.23 a	2.93 ± 1.14 c	2.69 ± 0.21 c
5-Hydroxy-6-methoxycoumarin 7-glucoside	0.44 ± 0.07 b	0.33 ± 0.06 b	0.39 ± 0.16 b	1.04 ± 0.05 a	0.22 ± 0.07 b	0.30 ± 0.04 b
Chlorogenic acid (3-CQA)	24.78 ± 0.14 b	23.51 ± 0.50 c	16.90 ± 0.17 e	35.14 ± 0.45 a	20.10 ± 0.45 d	23.10 ± 0.47 c
Eriodictyol 7-O-neohesperidoside (Neoeriocitrin)	0.01 ± 0.00 a	0.01 ± 0.00 a	0.01 ± 0.00 a	0.00 ± 0.00 a	0.00 ± 0.01 a	0.01 ± 0.01 a
Caffeic acid	0.25 ± 0.05 b	0.30 ± 0.02 a	0.14 ± 0.02	0.27 ± 0.04 a	0.17 ± 0.05 c	0.28 ± 0.04 a
Quercetin 3-glucosyl-(1->2)-galactoside	0.01 ± 0.01 a	0.01 ± 0.00 a	0.01 ± 0.00 a	0.01 ± 0.00 a	0.01 ± 0.00 a	0.02 ± 0.00 a
Quercetin-3-O-rutinoside (Rutin)	0.01 ± 0.01 a	0.01 ± 0.00 a	0.01 ± 0.01 a	0.01 ± 0.00 a	0.01 ± 0.00 a	0.02 ± 0.00 a
Quercetin 3-galactoside	0.16 ± 0.04 ab	0.09 ± 0.02 b	0.07 ± 0.02 b	0.05 ± 0.01 b	0.03 ± 0.01 c	0.24 ± 0.05 a
1,3-Dicaffeoyquinic acid (1,3-diCQA)	30.79 ± 0.70 b	23.79 ± 0.29 c	19.37 ± 0.82 d	31.97 ± 0.26 a	16.03 ± 0.78 e	26.83 ± 0.28 c
1,4-Dicaffeoyquinic acid (1,4-diCQA)	12.95 ± 0.80 b	4.93 ± 0.16 c	5.76 ± 0.39 c	21.40 ± 0.93 a	5.93 ± 3.01 c	13.27 ± 0.31 b
3,5-Dicaffeoyquinic acid (3,5-diCQA)	31.52 ± 0.17 b	21.00 ± 0.47 d	10.02 ± 0.56 f	36.77 ± 0.63 a	16.57 ± 8.30 e	26.83 ± 0.39 c
4,5-Dicaffeoyquinic acid (4,5-diCQA)	0.70 ± 0.11 a	0.39 ± 0.08 d	0.28 ± 0.03 d	0.86 ± 0.08 a	0.43 ± 0.03 c	0.62 ± 0.07 b

Means followed by the same letter within the row are not significantly different at *p* < 0.05.

**Table 2:** Comparison of antioxidant properties in the storage roots of Southern African sweet potato (*Ipomoea batatas* L.) varieties with Beauregard from the USA and Peruvian 199062.1.

	Orange Fleshed Storage Roots			Cream Fleshed Roots		
	‘Bophelo’	‘Beauregard’	‘199062.1′	‘Monate’	‘Ndou’	‘Blesbok’
FRAP (µM TEAC g^−1^)	525.63 ± 1.09 b	459.53 ± 1.84 c	532.06 ± 7.87 a	184.20 ± 1.51 f	231.58 ± 3.27 e	272.82 ± 1.51 d
DPPH (IC_50_ mg mL^−1^)	4.27 ± 0.10 b	4.42 ± 0.03 b	4.60 ± 0.03 b	6.39 ± 0.02 a	6.05 ± 0.10 a	6.80 ± 0.02 a
ABTS (IC_50_ mg mL^−1^)	4.32 ± 0.20 b	4.32 ± 0.20 b	4.73 ± 0.5 b	6.67 ± 0.04 a	6.12 ± 0.30 a	6.90 ± 0.12 a

Means followed by the same letter within the row are not significantly different at *p* < 0.05.

## Data Availability

Data is contained within the article or supplementary material.

## Supplementary Materials

The following supporting information can be downloaded at: https://www.mdpi.com/article/10.3390/foods11091329/s1, Figure S1: ESI negative mode BPI chromatogram of metabolites observed in the storage root of six different sweet potato varieties, Table S1: The calibration standard curve equations and the LOD and LOQ values.
