# Supplementary material for: Comparison of Caffeoylquinic Acids and Functional Properties of Domestic Sweet Potato (Ipomoea batatas (L.) Lam.) Storage Roots with Established Overseas Varieties
Source: Foods. 2022 May 3;11(9):1329. doi: 10.3390/foods11091329 (PMC9104689; doi:10.3390/foods11091329)
Supplement: Supplementary file 1 [file foods-11-01329-s001.zip › foods-1651471-supplementary.pdf]

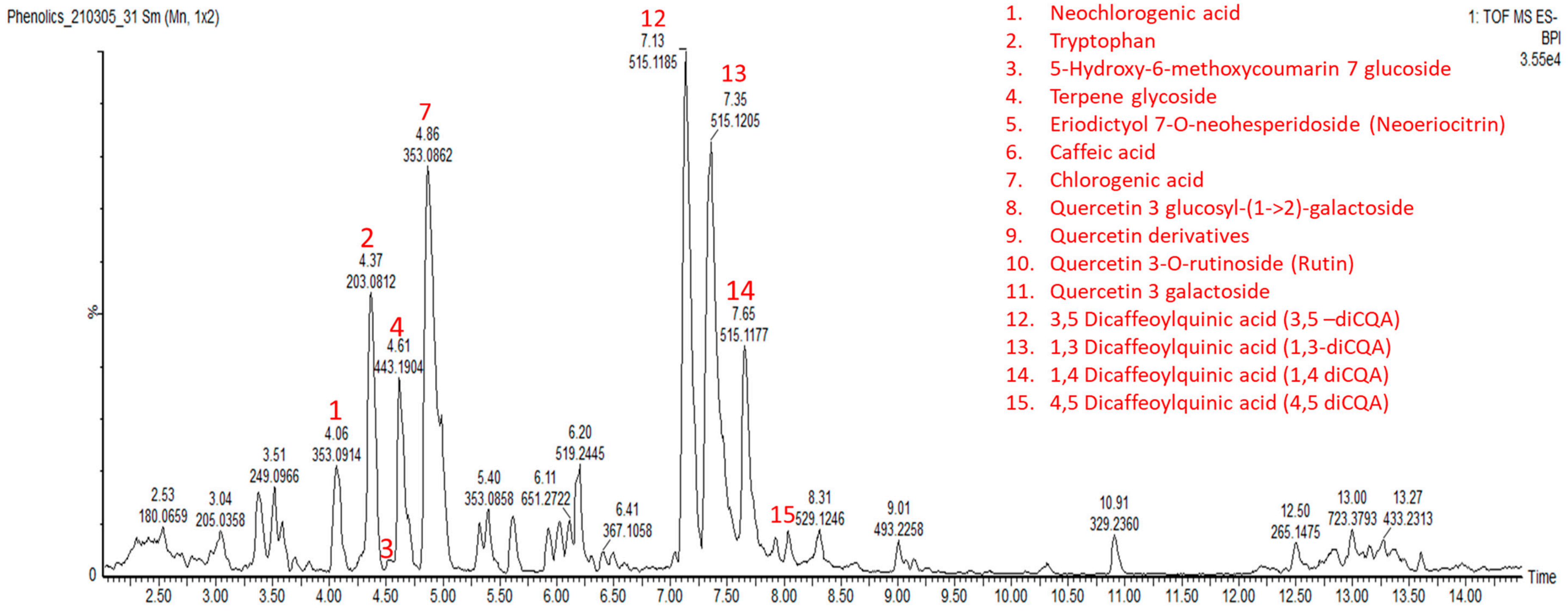

Supplementary Figure S1: ESI negative mode BPI chromatogram of metabolites observed in the storage root of six different sweet potato varieties.

Supplementary Table S1. The calibration standard curve equations and the LOD and LOQ values.

Standard curve	Equation	R^2	LOD (µg/ml)	LOQ (µg/ml)
Chlorogenic acid (spectrophotometer) for Total phenols	Y=0.0028x + 0.0428	0.98	16.35	49.54
catechin	Y=2828x-69172	0.99	3.2	10.9
rutin	Y=35316x+193517	0.997	1.4	3.3
Chlorogenic acid	Y=71930x-2110,5	0.995	0.11	0.37
			LOD (mM)	LOQ mM
Trolox	Y=0.3250x + 0.009	0.999	0.0058	0.0178
